# Host Phylogeny Shapes Gut Microbiota and Predicted Functions in Captive Artiodactyls

**DOI:** 10.3390/microorganisms13102250

**Published:** 2025-09-25

**Authors:** Guolei Sun, Tian Xia, Qinguo Wei, Xibao Wang, Yuehuan Dong, Xiufeng Yang, Lei Zhang, Weilai Sha, Honghai Zhang

**Affiliations:** College of Life Sciences, Qufu Normal University, Qufu 273165, China; sunguolei1989@163.com (G.S.); qfxiatian1993@163.com (T.X.); qgwei2008@163.com (Q.W.); wangxibao1995@163.com (X.W.); dongyuehuan2019@163.com (Y.D.); yangxf9066@163.com (X.Y.); zhanglei309418@163.com (L.Z.); shaweilai@163.com (W.S.)

**Keywords:** captive artiodactyls, gut microbiota, phylosymbiosis, host phylogeny, 16S rRNA amplicon sequencing

## Abstract

Host phylogeny can imprint the gut microbiota, but it is often masked by diet and environment. Leveraging the standardized husbandry of a zoological collection, we profiled fecal microbiota from 55 captive artiodactyls representing 12 species in Bovidae, Cervidae, and Camelidae using 16S rRNA amplicon sequencing targeting the V3–V4 region on the Illumina MiSeq platform. Community composition differed significantly among host families (Bray–Curtis PERMANOVA, R^2^ = 0.1075, *p* = 0.001). A host–microbiota tanglegram, which juxtaposes the host phylogeny with a dendrogram of microbiota similarity, recovered a topology congruent with the host phylogeny, with camelids forming a distinct branch separate from true ruminants in both trees. The linear discriminant analysis effect size (LEfSe; LDA ≥ 3.5) identified family-specific biomarkers, including enrichment of *Acinetobacter*/Moraxellaceae in Bovidae, Rikenellaceae (the *Rikenellaceae_RC9_gut_group*) in Cervidae, and *Rummeliibacillus* together with the *Christensenellaceae_R-7_group* in Camelidae. Functional inference with PICRUSt2 revealed significant differences in KEGG level-2 pathways (e.g., carbohydrate metabolism and xenobiotics biodegradation), consistent with taxonomic shifts. Altogether, these findings show that—even under uniform captive conditions—host evolutionary history remains a primary determinant of both the structure and the predicted functions of the artiodactyl gut microbiota, refining the scope of phylosymbiosis and providing actionable baselines for veterinary monitoring and conservation management in zoo settings.

## 1. Introduction

The gut microbiota—often termed the host’s “second genome”—supports nutrition, immune training, and defense against pathogens, thereby influencing health across mammals [1]. These roles are particularly critical for herbivores. Artiodactyla, one of the most diverse orders of herbivorous mammals, depends on dense microbial consortia to ferment structural polysaccharides into short-chain fatty acids and to synthesize essential vitamins and amino acids [2,3,4]. In this study, “host groups” specifically refer to three taxonomic families within Artiodactyla—Bovidae and Cervidae (true ruminants) and Camelidae (camelids/pseudoruminants)—which differ in digestive physiology and may therefore harbor distinct gut communities.

The assembly of mammalian gut communities is shaped by both extrinsic and intrinsic forces. Diet is frequently the most immediate external driver of community structure [5]. However, a growing body of evidence indicates that host genetics and evolutionary history impose a more fundamental and stable filter on microbiome assembly [6]. To explain this pattern, the concept of phylosymbiosis was proposed: microbial community similarity should increase with host phylogenetic relatedness, such that closely related hosts harbor more similar microbiotas than distant relatives [7,8]. Support for phylosymbiosis has accumulated across diverse animal lineages, including insects, fishes, and primates, although the signal often weakens when diet and environment vary widely [9,10,11]. Disentangling host-intrinsic effects from these confounders remains central to understanding host–microbe co-evolution [12].

Artiodactyls provide a compelling system for such tests because they encompass multiple families that differ in digestive anatomy and strategy (e.g., true ruminants versus pseudoruminant camelids). Nevertheless, broad phylogeny-wide evaluations across families remain limited, and field studies—while ecologically realistic—can obscure host-genetic signals due to heterogeneous diets and habitats [2,3,4,6,12]. For instance, Bovidae and Cervidae are both true ruminants but differ in their relative reliance on grazing versus browsing, while Camelidae, though also foregut fermenters, are not true ruminants and exhibit distinct digestive adaptations. Such ecological differences can blur phylogenetic patterns in natural settings. Zoological collections offer a practical solution by acting as “natural laboratories”: species are maintained under comparable conditions and standardized husbandry, enabling the internal signal of host phylogeny to be assessed with minimal interference from major external drivers [13,14].

Here, we leverage this framework to examine phylosymbiosis in captive artiodactyls. Using 16S rRNA gene amplicon sequencing, we profiled the fecal microbiota of 55 individuals representing 12 species across three families—Bovidae, Cervidae, and Camelidae—kept at a single zoological garden (Jinan Zoo, Shandong Province, China). We asked whether, under uniform husbandry, host family (and species) remains the primary determinant of gut-microbiota structure and whether predicted functional profiles mirror the observed taxonomic patterns. Specifically, we (i) compared alpha and beta diversity among host families, (ii) tested the congruence between the host phylogeny and microbiota clustering using a tanglegram analysis, (iii) identified family-specific microbial biomarkers with LEfSe, and (iv) inferred functional potentials with PICRUSt2 to evaluate differences in KEGG pathways. We hypothesized that host phylogeny—at least at the family level—would still drive both community structure and predicted functions despite standardized diet and environment. Beyond advancing the understanding of host–microbe co-evolution in herbivores, establishing family-specific baselines has immediate value for veterinary microbiome monitoring and conservation management of captive ungulates [15,16].

## 2. Materials and Methods

### 2.1. Sample Collection

Fresh fecal samples were collected in April 2017 at Jinan Zoo (Jinan, China). In total, 55 samples were obtained from 12 artiodactyl species spanning three families—Cervidae, Bovidae, and Camelidae (see Table 1). All sampled animals were visually healthy adults and had not received antibiotics or other microbiota-modulating drugs for at least three months prior to sampling. Animals were maintained under uniform husbandry and a standardized diet to minimize environmental and dietary variation. The standardized diet consisted of commercial herbivore pellets, fresh forage (e.g., alfalfa hay, seasonal vegetables), and ad libitum access to water, and had been consistently maintained for at least one year prior to sampling. According to veterinary records, all sampled animals were healthy adults with no exposure to antibiotics or other microbiota-modulating drugs (including anthelmintics or corticosteroids) for at least three months before sampling; individuals not meeting these criteria were excluded.

Immediately after defecation, feces were sampled from the mid-section of each bolus, thoroughly homogenized, aliquoted into sterile cryotubes, and flash-frozen in liquid nitrogen on site. Samples were then transported to the laboratory on dry ice within 48 h and stored at −80 °C until DNA extraction. Sampling was non-invasive and conducted in accordance with institutional and national guidelines; procedures were reviewed and approved by the Qufu Normal University Institutional Animal Care and Use Committee (IACUC) (Permit Number: QFNU2017-014).

This study was designed as an observational comparison under standardized zoo husbandry. Species were chosen to span three phylogenetically distinct families (Bovidae, Cervidae, and Camelidae) represented at the institution; when available, 4–5 healthy adults were sampled per species, and final numbers reflected the availability of eligible animals (total *n* = 55). Because the number of animals per species was limited, strict randomization was not feasible; therefore, a census-style sampling strategy was adopted in which all eligible individuals were included. To minimize potential bias, all samples were handled and processed using the same standardized laboratory protocols.

### 2.2. DNA Extraction and 16S rRNA Gene Sequencing

Total genomic DNA was extracted from ~200 mg feces using the QIAamp DNA Stool Mini Kit (Qiagen, Hilden, Germany) according to the manufacturer’s instructions. The V3–V4 hypervariable region of the bacterial 16S rRNA gene was amplified with primers 341F (5′-CCTACGGGNGGCWGCAG-3′) and 806R (5′-GGACTACHVGGGTWTCTAAT-3′) [17]. PCR products were purified, quantified, and pooled in equimolar amounts to construct sequencing libraries. Paired-end sequencing (2 × 250 bp) was performed on an Illumina MiSeq/PE250 platform at Genedenovo Biotechnology Co., Ltd. (Guangzhou, China) in December 2021.

### 2.3. Bioinformatic Processing

Raw reads were quality-filtered with fastp (v0.18.0) and merged into tags with FLASH (v1.2.7) [18,19]. High-quality tags were clustered into operational taxonomic units (OTUs) at 97% sequence identity using the UPARSE pipeline in USEARCH (v7.0) with chimera removal enabled [20]. Representative sequences of each OTU were taxonomically assigned using the RDP Classifier (v2.2) against the SILVA reference database (v138) [21]. Unless otherwise stated, downstream analyses used the resulting OTU table.

### 2.4. Statistical and Ecological Analyses

All statistical analyses were performed in R (v4.0.3). For alpha diversity, we calculated Chao1 (richness), Shannon (diversity), and Faith’s phylogenetic diversity (PD) for each sample. Differences among host families were evaluated using the Kruskal–Wallis test, followed—where appropriate—by pairwise Dunn tests with Benjamini–Hochberg false-discovery-rate correction. For beta diversity, community dissimilarities were computed with Bray–Curtis distances and visualized by principal coordinates analysis (PCoA); among-group differences were tested with PERMANOVA (adonis, based on permutation testing) and corroborated with ANOSIM in the vegan package [22].

To identify differential taxa, we applied LEfSe with α = 0.05 (Kruskal–Wallis) and an LDA score ≥ 3.5 [23]. To assess phylosymbiosis, OTU relative abundances were averaged within each host species, Bray–Curtis distances were calculated, and an average-linkage (UPGMA) microbiota dendrogram was constructed. The host phylogeny was obtained from divergence times in TimeTree (https://timetree.org/, accessed on 10 August 2025) [24], and a tanglegram, which visually compares the host phylogeny with the microbiota similarity dendrogram, was plotted in R to test the congruence between evolutionary history and microbial community structure.

### 2.5. Functional Prediction

Functional potentials were inferred using PICRUSt2 (v2.3.0-b) [25]. Predicted genes were mapped to KEGG orthologs and summarized at pathway level 2 (level 3 where indicated) [26]. Differences among host families were assessed using non-parametric tests in R (Kruskal–Wallis with Benjamini–Hochberg correction).

## 3. Results

### 3.1. Sequencing Data Overview

**Figure 1:**
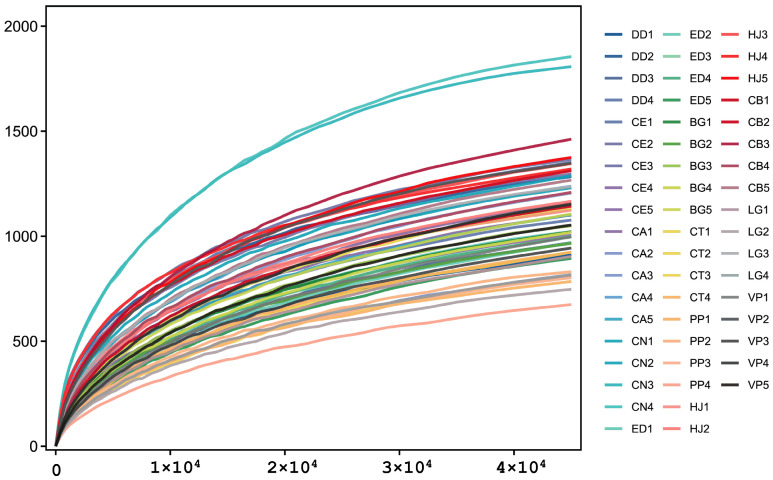
Rarefaction (sobs) curves of the 55 fecal samples. Each curve represents one sample; curves approach a plateau, indicating adequate sequencing depth. Sample IDs consist of a two-letter species code followed by an individual number. Species codes: DD = *Dama dama*, CE = *Cervus elaphus*, CA = *Cervus albirostris*, CN = *Cervus nippon*, ED = *Elaphurus davidianus*, BG = *Bos grunniens*, CT = *Connochaetes taurinus*, PP = *Procapra przewalskii*, HJ = *Hemitragus jemlahicus*, CB = *Camelus bactrianus*, LG = *Lama guanicoe*, VP = *Vicugna pacos*. (Family groups: Cervidae = DD, CE, CA, CN, ED; Bovidae = BG, CT, PP, HJ; Camelidae = CB, LG, VP).

Across the 55 fecal samples, high-throughput sequencing of the 16S rRNA V3–V4 region generated 7,117,942 raw reads. After quality control, merging and chimera removal, 6,089,737 high-quality tags were retained, averaging 110,722 per sample, with an effective rate of 85.55% (Table S1). At a 97% similarity threshold, sequences clustered into 69,041 OTUs; per-sample OTU counts ranged from 815 to 1869 (mean 1255; Table S1). Rarefaction curves approached saturation for all samples, and Good’s coverage exceeded 99.5%, indicating adequate depth for downstream analyses (Figure 1 and Figure S1).

### 3.2. Host Family Shaped Overall Diversity and Structure

**Figure 2:**
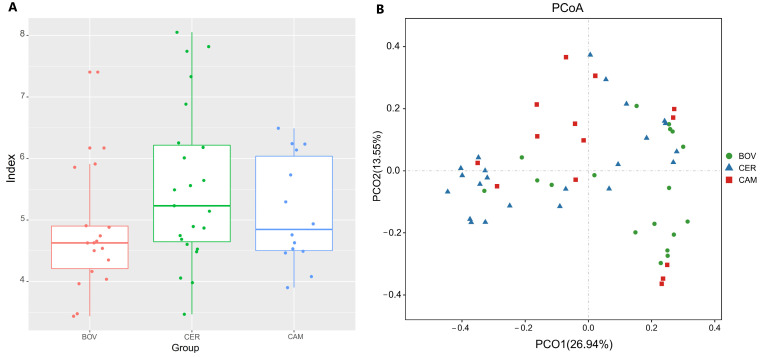
Diversity by host family. (**A**) Alpha-diversity indices across Bovidae (BOV), Cervidae (CER), and Camelidae (CAM) (Faith’s PD differed, while richness/diversity were broadly comparable). (**B**) PCoA on Bray–Curtis dissimilarities indicated partial separation with some overlap among families (PC1 = 26.94%, PC2 = 13.55%). This visual trend was strongly supported by PERMANOVA (R*^2^* = 0.1075, *p* = 0.001) and further confirmed by ANOSIM (R = 0.157, *p* = 0.001; Figure S3).

Alpha-diversity comparisons among Bovidae (BOV), Cervidae (CER) and Camelidae (CAM) revealed no significant differences in richness (sobs, Chao1, ACE) or diversity (Shannon, Simpson) (Figure 2A and Figure S2; Kruskal–Wallis, *p* > 0.05). By contrast, phylogenetic diversity (PD) differed among families (Kruskal–Wallis, *p* = 0.012; Table S2), suggesting detectable phylogenetic diversification of communities.

For beta diversity, PCoA based on Bray–Curtis dissimilarities indicated partial separation with some overlap among families (PC1 26.94%, PC2 13.55%; Figure 2B). PERMANOVA supported significant among-family differences (R^2^ = 0.1075, *p* = 0.001; Table S3). While this R^2^ value is modest, it represents a biologically meaningful effect size in the context of a complex ecosystem, indicating that host family is a robust driver of community structure. This conclusion was corroborated by ANOSIM (R = 0.157, *p* = 0.001; Figure S3), and the pattern was also consistent in NMDS. Similar separations were further observed using Jaccard, unweighted UniFrac, and weighted UniFrac distances (Figure S4). Together, these results indicate that host family is a major determinant of overall community structure.

### 3.3. Community Composition Mirrored Host Phylogeny

**Figure 3:**
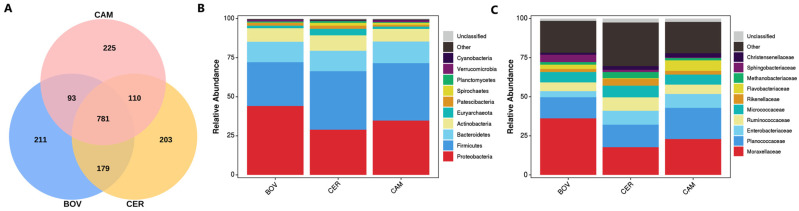
Community composition across host families. (**A**) Venn diagram of shared and unique OTUs among the three families (core present in all). (**B**) Phylum-level profiles were dominated by Proteobacteria, Firmicutes and Bacteroidetes, with family-specific shifts. (**C**) At finer ranks, Moraxellaceae was the highest in Bovidae and Camelidae, Planococcaceae was common across families, and *Acinetobacter* was prominent overall.

A three-way Venn diagram identified a core set of 781 OTUs shared by all three families, alongside numerous family-specific OTUs (BOV 211, CER 203 and CAM 225), indicating both conserved and lineage-specific microbiome members (Figure 3A). At the phylum level, communities were dominated by Proteobacteria, Firmicutes and Bacteroidetes (>79% in total), but their relative abundances shifted among families: bovids were dominated by Proteobacteria (mean 44.07%), whereas cervids (37.45%) and camelids (36.70%) were Firmicutes-dominant; Bacteroidetes remained comparatively stable (13.01–13.87%) (Figure 3B).

At finer ranks, Moraxellaceae was the top family in Bovidae (36.12%) and Camelidae (22.94%), but lower in Cervidae (17.68%); Planococcaceae was abundant across all families, especially in Camelidae (19.92%) (Figure 3C). At the genus level, *Acinetobacter* was prominent overall and particularly enriched in Bovidae (mean 36.08%), while *Solibacillus* was relatively higher in Camelidae (10.39%) and Cervidae (7.55%) than in Bovidae (4.69%) (Figure 3C). These shifts provide a taxonomic basis for the family-level separation seen in beta-diversity analyses.

### 3.4. Family-Specific Biomarkers Identified by LEfSe

**Figure 4:**
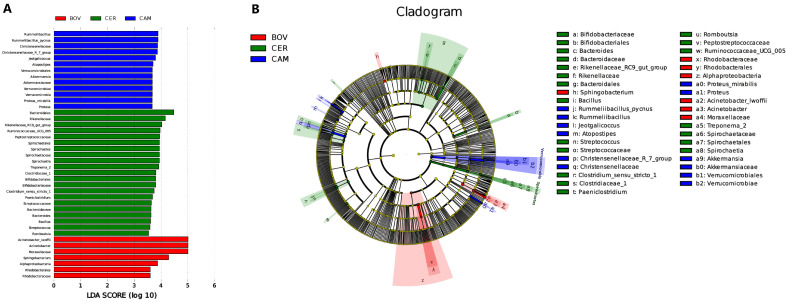
LEfSe biomarkers by host family. (**A**) LDA score (log10) bar plot (α = 0.05; LDA ≥ 3.5). (**B**) Cladogram of significant taxa; colors denote BOV (red), CER (green), CAM (blue). Exemplary markers: *Acinetobacter* (Moraxellaceae) (BOV), Rikenellaceae and the Rikenellaceae_RC9_gut_group (CER), *Ruminilibacillus* and the Christensenellaceae_R-7_group (CAM).

Using a stringent LEfSe threshold (α = 0.05, LDA ≥ 3.5), we detected distinct microbial fingerprints for each host family (Figure 4; Table S4). Bovidae were characterized by *Acinetobacter* (family Moraxellaceae); Cervidae showed enrichment of Rikenellaceae; Camelidae featured *Ruminilibacillus* together with the Christensenellaceae_R-7_group; Camelidae featured *Rummeliibacillus* and the Christensenellaceae_R-7_group. Even under strict filtering, the three families exhibited clear and non-overlapping biomarker sets.

### 3.5. Direct Test of Phylosymbiosis with a Host–Microbiota Tanglegram

**Figure 5:**
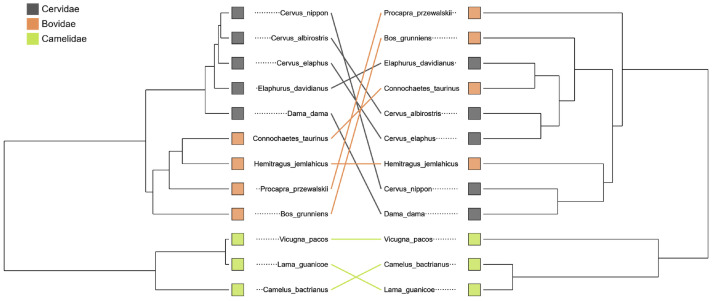
Host–microbiota tanglegram. Species-averaged communities (Bray–Curtis, UPGMA) are compared with the TimeTree host phylogeny; camelids form a distinct, conserved branch, whereas crossings mainly occur between bovids and cervids.

To visualize congruence between host evolution and microbiota structure, we averaged OTU abundances within species and built an UPGMA dendrogram from Bray–Curtis distances and compared it with the host phylogeny from TimeTree using a tanglegram (Figure 5). The two trees exhibited clear topological agreement: camelids formed an independent and highly conserved branch in both trees. In contrast, relationships between Bovidae and Cervidae were more complex at this shallower phylogenetic scale, with several crossing links, suggesting that while the phylosymbiosis signal is strong at the family level, it can weaken among more closely related families.

### 3.6. PICRUSt2-Inferred Functions Varied with Host Family

**Figure 6:**
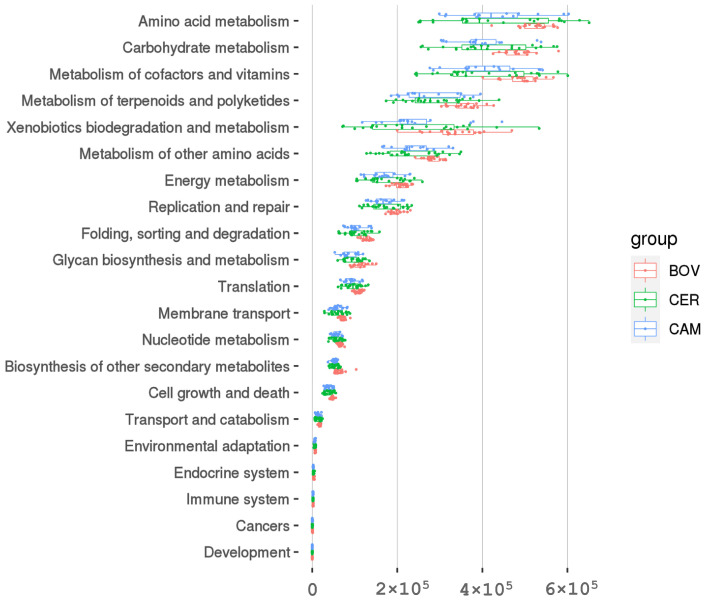
PICRUSt2-inferred KEGG level-2 functions. Relative abundances of functional pathways across the three families; Metabolism dominated (level 1), with Amino acid metabolism, Carbohydrate metabolism, and Metabolism of cofactors and vitamins among the most abundant at level 2, and several pathways differing among families.

Predicted functional profiles (PICRUSt2) were dominated by metabolic functions (KEGG level 1) across all families. At level 2, the most abundant pathways included Amino acid metabolism, Carbohydrate metabolism, and Metabolism of cofactors and vitamins (Table S5). Multiple level-2 pathways differed significantly among families (Kruskal–Wallis, *p* < 0.05), including higher Carbohydrate metabolism in Cervidae relative to Camelidae, as well as differences in Xenobiotics biodegradation and metabolism, Metabolism of other amino acids, and Glycan biosynthesis and metabolism (Figure 6; Table S5). Thus, taxonomic shifts among host families were accompanied by corresponding differences in predicted metabolic potentials.

## 4. Discussion

Under tightly standardized husbandry, host lineage still left a clear imprint on the gut microbiota of captive artiodactyls, providing direct support for phylosymbiosis. In natural settings, dietary and environmental heterogeneity can blur or even override host signals, complicating efforts to isolate intrinsic host effects [14,27]. By using a zoo as a “natural laboratory”—that is, a controlled setting where diet and environment are standardized across species—our design minimized these exogenous drivers and positioned host lineage (family level) as the primary variable [5,28,29]. Multiple, mutually reinforcing lines of evidence—ordination based on Bray–Curtis, significance of the among-family term in PERMANOVA, distinct LEfSe biomarker sets, and the concordant host–microbiota tanglegram—converged on the same conclusion that host evolutionary history is a fundamental internal driver of community structure [6,7,13]. Methodologically, this agreement across dissimilarity metrics and analytical frameworks increases confidence that the observed pattern is not an artifact of a single pipeline but rather a stable feature of the data, consistent with broader evidence for host–microbe coupling in mammals [30]. Notably, alpha-diversity indices of richness and evenness were broadly comparable among families, whereas phylogenetic diversity (PD) differed, suggesting that host effects were expressed less as simple gains or losses of taxa and more as lineage-specific rearrangements along the phylogenetic tree—an outcome expected when long-term host filters act on clade membership rather than total OTU counts.

The tanglegram captured a macro-evolutionary pattern that mirrors a major split in artiodactyl evolution: camelids (Tylopoda) versus true ruminants (Ruminantia) [31,32]. Camelids possess a three-chambered fore-stomach, longer digesta retention, and physiological adaptations to arid environments; these features likely impose persistent selection on gut consortia and the functions they supply [33,34]. In line with this interpretation, Camelidae were characterized by Christensenellaceae (including the Christensenellaceae_R-7_group), together with *Ruminilibacillus*, whose representatives participate in specialized metabolic processes [3,35,36,37]. The high topological congruence of camelids across the paired host and microbiota trees therefore points to deep co-adaptation between host physiology and microbial functions, offering a visually intuitive instance of phylosymbiosis at a macroevolutionary scale [7,11].

At shallower evolutionary scales within Ruminantia, the picture was more nuanced. Crossing links between Bovidae and Cervidae in the tanglegram indicate that closely related families do not always display perfectly parallel microbiota topologies [14,38,39]. A biological interpretation is that long-standing differences in foraging strategies and digestive anatomy—many bovids tending toward grazing and many cervids toward browsing, with attendant contrasts in particle size reduction, salivary buffering, and rumen compartment use—shape distinct ecological niches for microbial guilds that persist even under a uniform captive diet [40,41]. In this context, our LEfSe results are coherent: Bovidae were marked by *Acinetobacter* (Moraxellaceae), a genus frequently detected in herbivore guts and noted for metabolic versatility, whereas Cervidae showed enrichment of Rikenellaceae (notably the Rikenellaceae_RC9_gut_group), taxa commonly associated with plant-polysaccharide turnover in herbivores [42,43,44]. Rather than contradicting phylosymbiosis, these patterns suggest that phylogenetic signal is partly modulated by lineage-typical feeding ecologies at this shallow scale, producing family-specific “modules” within a shared herbivore meta-community.

Predicted functions echoed the taxonomic shifts and help contextualize them mechanistically. PICRUSt2 indicated among-family differences at KEGG level 2, including carbohydrate metabolism and xenobiotics biodegradation. The former is consistent with variable reliance on fiber fermentation pathways across families, whereas the latter plausibly reflects differential handling of plant secondary compounds typical of grazer- versus browser-biased diets. Differences in “metabolism of other amino acids” and “glycan biosynthesis and metabolism” further imply reconfiguration of cell-wall and storage-polysaccharide pathways within the microbiota. Together with largely comparable richness/evenness but distinct phylogenetic diversity, these results fit a scenario of functional complementarity—host lineages assemble alternative taxonomic consortia to deliver overlapping but not identical metabolic capacities [29,45]. Beyond evolutionary inference, such lineage-informed baselines have immediate practical value in zoos: non-invasive fecal profiling can flag early deviations from family-typical configurations that may presage dysbiosis or stress [15,46,47], and knowledge of lineage-biased fermentation guilds can guide diet formulation and conservation management for threatened ungulates under ex situ programs [48,49].

Several limitations merit caution. First, 16S rRNA amplicons limit taxonomic resolution, and PICRUSt2 yields predicted rather than measured functions [50]. Second, captivity itself can alter gut diversity and structure, constraining direct extrapolation to wild populations [51]. Although unreported treatments cannot be completely ruled out, the ≥3-month medication-free criterion and verification against veterinary records make residual confounding by medication unlikely under the study conditions. We acknowledge that we did not sub-sample multiple parts of a single fecal bolus; although homogenization reduces intra-sample variation, this remains a limitation for future validation. Moreover, extrapolation of our conclusions from controlled zoo conditions to wild populations should be made with caution, and future comparative studies with wild counterparts are needed. Future work should therefore (i) apply shotgun metagenomics and metabolomics to validate pathway differences at gene and metabolite levels [52,53]; (ii) isolate and test marker taxa—e.g., cervid-enriched Rikenellaceae—in targeted substrate-use assays to establish mechanism; and (iii) include wild or semi-wild cohorts with longitudinal sampling to separate genetic signals from captivity-driven effects. Alongside the present evidence that host lineage exerts a detectable main effect under minimized external noise [54,55], these steps will move the field from comparative patterns toward mechanism and application—potentially enabling tailored prebiotics or probiotics for vulnerable artiodactyls in ex situ conservation.

## 5. Conclusions

In this study, we demonstrate that host phylogeny remains the dominant force shaping the composition and predicted functions of the artiodactyl gut microbiota under highly standardized zoo conditions. This lineage-informed signal provides robust, family-level microbial baselines that zoos can use for non-invasive health monitoring, targeted diet optimization and conservation breeding, enabling curators to detect early deviations from lineage norms that may indicate stress, disease or nutritional imbalance. Looking ahead, the next steps are to move from patterns to mechanisms by validating functions with shotgun metagenomics, metatranscriptomics, metabolomics and in vitro fermentations; to test generality through coordinated multi-zoo and wild-cohort comparisons; and to broaden scope by integrating the mycobiome and virome. Together, these directions will refine the evolutionary framework revealed here and translate it into scalable tools for evidence-based management and research in microbial ecology and wildlife conservation.

## Figures and Tables

**Table 1:** Information on the artiodactyl species sampled in this study.

Common Name	Scientific Name	Family	No. of Samples (*n*)
Fallow deer	*Dama dama*	Cervidae	4
Red deer	*Cervus elaphus*	Cervidae	5
White-lipped deer	*Cervus albirostris*	Cervidae	5
Sika deer	*Cervus nippon*	Cervidae	4
Père David’s deer	*Elaphurus davidianus*	Cervidae	5
Domestic yak	*Bos grunniens*	Bovidae	5
Blue wildebeest	*Connochaetes taurinus*	Bovidae	4
Przewalski’s gazelle	*Procapra przewalskii*	Bovidae	4
Himalayan tahr	*Hemitragus jemlahicus*	Bovidae	5
Bactrian camel	*Camelus bactrianus*	Camelidae	5
Guanaco	*Lama guanicoe*	Camelidae	4
Alpaca	*Vicugna pacos*	Camelidae	5

Note: Scientific names are verified according to the Integrated Taxonomic Information System (ITIS) and NCBI Taxonomy. All fecal samples were collected at Jinan Zoo, China.

## Data Availability

Raw 16S rRNA amplicon reads have been deposited in the NCBI Sequence Read Archive (SRA) under BioProject PRJNA1305732 (Study SRP608857; https://www.ncbi.nlm.nih.gov/bioproject/PRJNA1305732, accessed on 14 August 2025). Individual runs are available under accessions SRR34987614–SRR34987668. Each run corresponds to one sample with paired FASTQ files (R1/R2).

## Supplementary Materials

The following supporting information can be downloaded at: https://www.mdpi.com/article/10.3390/microorganisms13102250/s1, Figure S1: Rank–abundance curves and Good’s coverage across 55 samples; Figure S2: Alpha-diversity indices (ACE, Chao1, Good’s coverage, Shannon, Simpson, Sobs and Faith’s PD) by host family; Figure S3: NMDS ordination and ANOSIM based on Bray–Curtis dissimilarities; Figure S4: PCoA using Jaccard, unweighted UniFrac and weighted UniFrac distances; Table S1: Sequencing throughput and quality-control summary; Table S2: Per-sample alpha-diversity indices; Table S3: PERMANOVA and ANOSIM among host families; Table S4: LEfSe biomarkers (LDA ≥ 3.5) with enriched family; Table S5: KEGG level-2 functional profiles (PICRUSt2) summarized by host family.
