# Supplementary material for: Host Phylogeny Shapes Gut Microbiota and Predicted Functions in Captive Artiodactyls
Source: Microorganisms. 2025 Sep 25;13(10):2250. doi: 10.3390/microorganisms13102250 (PMC12566460; doi:10.3390/microorganisms13102250)
Supplement: Supplementary file 1 [file microorganisms-13-02250-s001.zip › Supplementary_Materials.pdf]

Supplementary Materials (Figures & Tables)

Contents

Figure S1. Sequencing coverage and rank–abundance profiles across 55 samples.
Figure S2. Alpha-diversity indices by host family.
Figure S3. β-diversity based on Bray–Curtis dissimilarities.
Figure S4. Robustness of PCoA across distance metrics.
Table S1. Sequencing throughput and quality-control summary of fecal samples from captive artiodactyls.
Table S2. Per-sample alpha diversity indices for captive artiodactyls.
Table S3. PERMANOVA and ANOSIM results based on Bray–Curtis dissimilarity.
Table S4. LEfSe-derived microbial biomarkers (LDA ≥ 3.5, log10) across host families.
Table S5. KEGG Level 2 functional profiles (PICRUSt2) summarized by host family.

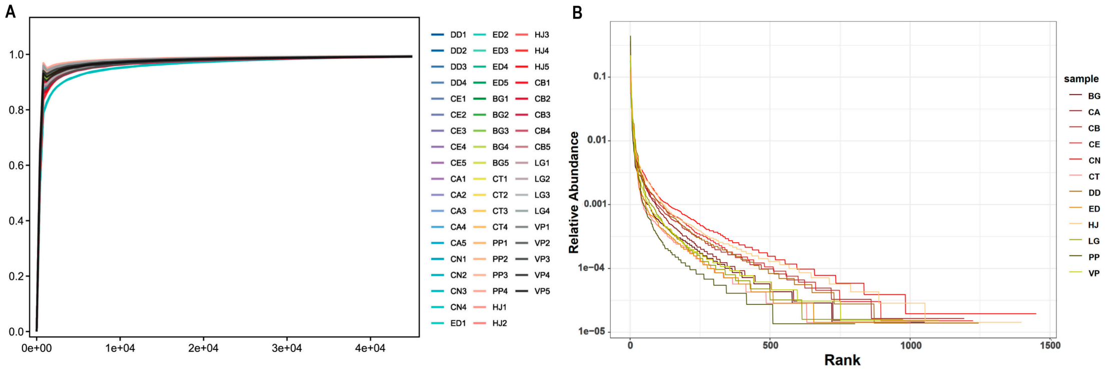

Supplementary Figure S1. Sequencing coverage and rank–abundance profiles across 55 samples.

(A) Good’s coverage curves for each sample; values approach 1.0 and plateau, indicating sufficient sequencing depth. (B) Whittaker rank–abundance plots summarized by host family; curve length reflects richness and slope reflects evenness; Whittaker rank–abundance plots summarized by host family.

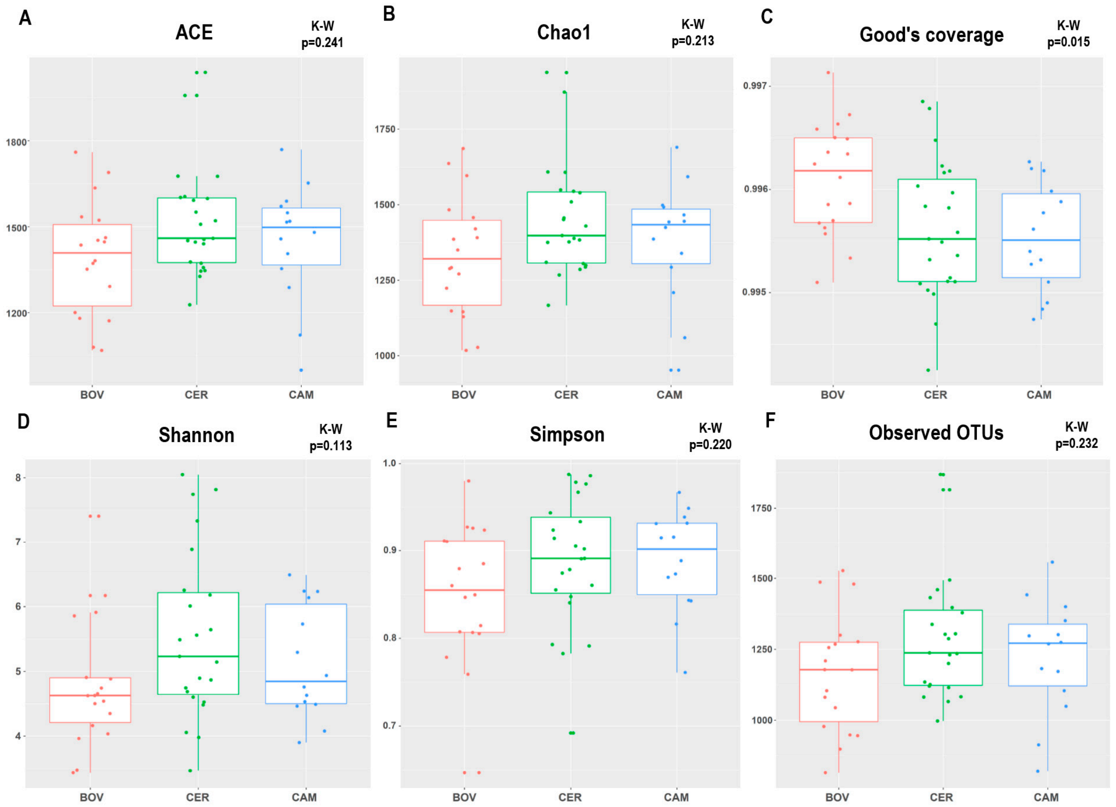

Supplementary Figure S2. Alpha-diversity indices by host family.

(A–F) Box plots of ACE, Chao1, Good’s coverage, Shannon, Simpson, and Sobs (observed OTUs) for Bovidae (BOV), Cervidae (CER), and Camelidae (CAM). Boxes show the interquartile range (IQR); center line is the median; whiskers extend to 1.5×IQR; outliers are shown as points.

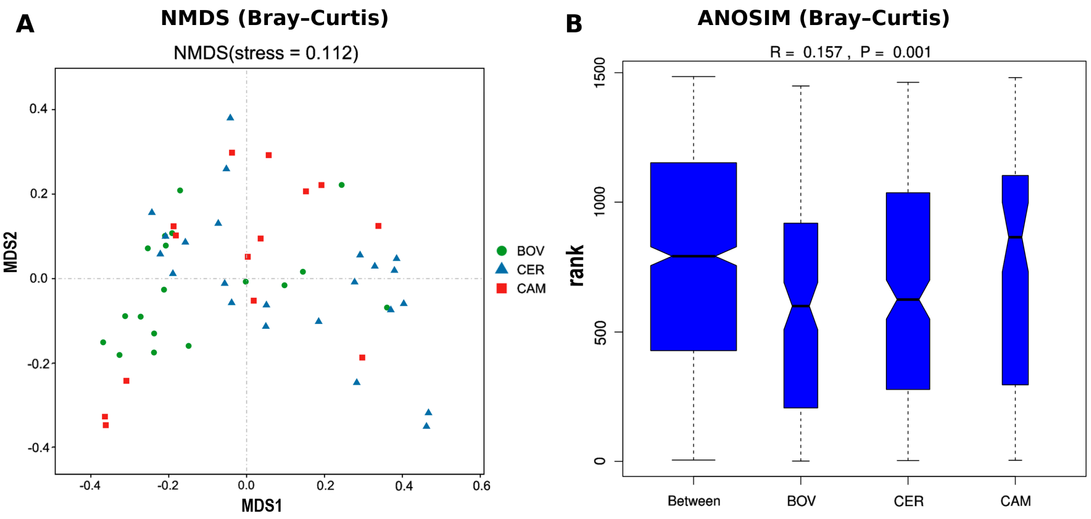

Supplementary Figure S3. β-diversity based on Bray–Curtis dissimilarities.

(A) NMDS ordination colored by host family (BOV, CER, CAM). (B) ANOSIM comparing within- versus between-family dissimilarities (R = 0.157, p = 0.001).

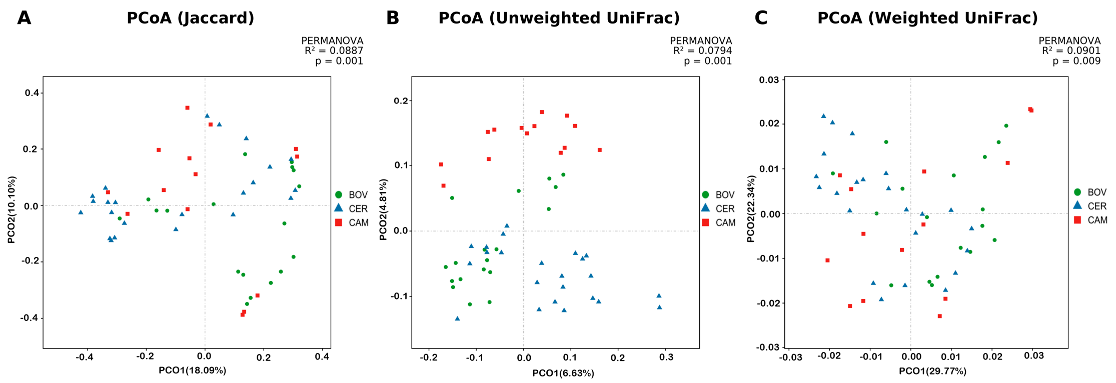

Supplementary Figure S4. Robustness of PCoA across distance metrics.

(A) Jaccard; (B) unweighted UniFrac; (C) weighted UniFrac. PERMANOVA statistics (R^2 and p) are shown in each panel; all metrics indicate significant among-family differences.

Table S1. Sequencing throughput and quality-control summary of fecal samples from captive artiodactyls.

Sample	Family	Species	Raw_ reads	Clean_ reads	Effective_ tags	Effective_ Ratio %	OTUs
DD1	Cervidae	Dama dama	131198	131046	115240	87.84	1081
DD2	Cervidae	Dama dama	137293	137101	119006	86.68	1432
DD3	Cervidae	Dama dama	128762	128606	111193	86.36	1120
DD4	Cervidae	Dama dama	126309	126110	107270	84.93	1460
CE1	Cervidae	Cervus elaphus	134025	133845	113541	84.72	1235
CE2	Cervidae	Cervus elaphus	131812	131633	114184	86.63	1494
CE3	Cervidae	Cervus elaphus	121534	121362	103736	85.36	1397
CE4	Cervidae	Cervus elaphus	125260	125075	106834	85.29	1287
CE5	Cervidae	Cervus elaphus	126271	126098	107720	85.31	1230
CA1	Cervidae	Cervus albirostris	124712	124562	104087	83.46	1083
CA2	Cervidae	Cervus albirostris	135771	135609	119513	88.03	997
CA3	Cervidae	Cervus albirostris	128922	128770	110747	85.9	1338
CA4	Cervidae	Cervus albirostris	120153	119975	100790	83.88	1303
CA5	Cervidae	Cervus albirostris	136052	135875	114917	84.47	1237
CN1	Cervidae	Cervus nippon	121010	120827	101756	84.09	1305
CN2	Cervidae	Cervus nippon	125971	125768	105826	84.01	1379
CN3	Cervidae	Cervus nippon	128829	128633	105371	81.79	1815
CN4	Cervidae	Cervus nippon	133409	133228	109524	82.1	1869
ED1	Cervidae	Elaphurus davidianus	137794	137611	119429	86.67	1200
ED2	Cervidae	Elaphurus davidianus	124403	124248	107105	86.1	1115
ED3	Cervidae	Elaphurus davidianus	124050	123867	106017	85.46	1126
ED4	Cervidae	Elaphurus davidianus	129176	129005	108867	84.28	1135
ED5	Cervidae	Elaphurus davidianus	137248	137083	119458	87.04	1066
BG1	Bovidae	Bos grunniens	133070	132866	115167	86.55	1178
BG2	Bovidae	Bos grunniens	122200	122026	103659	84.83	1081
BG3	Bovidae	Bos grunniens	120613	120460	104637	86.75	1209
BG4	Bovidae	Bos grunniens	132731	132555	114829	86.51	1277
BG5	Bovidae	Bos grunniens	132203	132044	112977	85.46	1178
CT1	Bovidae	Connochaetes taurinus	125015	124858	107664	86.12	1268
CT2	Bovidae	Connochaetes taurinus	127112	126941	109778	86.36	945
CT3	Bovidae	Connochaetes taurinus	137549	137365	118753	86.34	1104
CT4	Bovidae	Connochaetes taurinus	131713	131505	111278	84.49	1044
PP1	Bovidae	Procapra przewalskii	120415	120239	104800	87.03	898
PP2	Bovidae	Procapra przewalskii	129524	129325	112927	87.19	978
PP3	Bovidae	Procapra przewalskii	131042	130859	114534	87.4	948
PP4	Bovidae	Procapra przewalskii	120838	120666	106358	88.02	815
HJ1	Bovidae	Hemitragus jemlahicus	123476	123234	107589	87.13	1256
HJ2	Bovidae	Hemitragus jemlahicus	125283	125087	106981	85.39	1300
HJ3	Bovidae	Hemitragus jemlahicus	129495	129226	111700	86.26	1487
HJ4	Bovidae	Hemitragus jemlahicus	125735	125526	111857	88.96	1480
HJ5	Bovidae	Hemitragus jemlahicus	135617	135434	116954	86.24	1527
CB1	Camelidae	Camelus bactrianus	129613	129398	111892	86.33	1442
CB2	Camelidae	Camelus bactrianus	130230	130054	111757	85.82	1269
CB3	Camelidae	Camelus bactrianus	135333	135046	112675	83.26	1557
CB4	Camelidae	Camelus bactrianus	128426	128204	107140	83.43	1302
CB5	Camelidae	Camelus bactrianus	128336	128106	107308	83.61	1351
LG1	Camelidae	Lama guanicoe	132783	132532	115170	86.74	1049
LG2	Camelidae	Lama guanicoe	121823	121627	103546	85	820
LG3	Camelidae	Lama guanicoe	134800	134564	112132	83.18	1297
LG4	Camelidae	Lama guanicoe	136116	135905	115216	84.65	913

VP1	Camelidae	Vicugna pacos	136720	136469	117221	85.74	1172
VP2	Camelidae	Vicugna pacos	129356	129133	109575	84.71	1400
VP3	Camelidae	Vicugna pacos	131115	130879	113141	86.29	1104
VP4	Camelidae	Vicugna pacos	135853	135605	115179	84.78	1274
VP5	Camelidae	Vicugna pacos	133843	133631	113212	84.59	1182

Note: Effective ratio = (Effective_tags / Raw_reads) × 100.

Table S2. Per-sample alpha diversity indices for captive artiodactyls.

Sample	Sobs	Shannon	Simpson	Chao	Ace	Goods_cov (OTU)	Pielou	PD
BG1	1178	4.63	0.879	1292.0	1381.2	0.996	0.454	137.7
BG2	1081	4.66	0.847	1288.3	1372.0	0.995	0.462	133.4
BG3	1209	4.16	0.778	1385.5	1461.7	0.996	0.407	139.8
BG4	1277	4.54	0.814	1457.2	1522.2	0.996	0.44	143.8
BG5	1178	4.5	0.807	1349.7	1435.9	0.996	0.441	137.6
CT1	1268	4.74	0.86	1390.8	1452.4	0.996	0.46	143.6
CT2	945	4.63	0.911	1128.9	1179.8	0.996	0.468	123.2
CT3	1104	4.35	0.885	1270.5	1352.1	0.996	0.43	135.7
CT4	1044	4.88	0.926	1223.7	1291.4	0.996	0.487	129.2
HJ1	1256	5.86	0.927	1419.8	1447.4	0.996	0.569	135.3
HJ2	1300	4.91	0.85	1482.9	1534.2	0.996	0.474	138.8
HJ3	1487	6.17	0.924	1636.3	1689.2	0.996	0.586	152.8
HJ4	1480	7.4	0.98	1596.0	1634.9	0.997	0.703	136.1
HJ5	1527	5.91	0.911	1686.0	1760.0	0.995	0.559	160.1
PP1	898	3.44	0.647	1027.4	1079.2	0.997	0.35	109.0
PP2	978	4.04	0.806	1148.1	1200.1	0.997	0.406	121.2
PP3	948	3.97	0.805	1145.3	1171.0	0.997	0.401	113.3
PP4	815	3.48	0.759	1017.5	1068.1	0.997	0.359	95.7
CB1	1442	6.23	0.939	1592.6	1652.2	0.996	0.594	154.9
CB2	1269	4.94	0.869	1442.6	1515.8	0.995	0.479	147.8
CB3	1557	6.14	0.932	1690.0	1769.4	0.995	0.579	180.5
CB4	1302	5.29	0.915	1465.8	1548.7	0.995	0.512	144.9
CB5	1351	5.73	0.931	1492.0	1589.1	0.995	0.551	171.1
LG1	1049	4.53	0.873	1208.9	1287.5	0.996	0.452	137.5
LG2	820	4.63	0.916	952.1	999.0	0.996	0.478	112.2
LG3	1297	6.49	0.967	1386.2	1457.8	0.996	0.628	168.4
LG4	913	4.46	0.888	1059.8	1121.9	0.996	0.454	123.4
VP1	1172	4.08	0.816	1424.8	1480.4	0.995	0.4	137.4
VP2	1400	6.24	0.949	1498.0	1571.1	0.995	0.597	165.8
VP3	1104	3.9	0.761	1293.0	1353.7	0.996	0.386	132.1
VP4	1274	4.76	0.843	1445.3	1518.7	0.995	0.461	159.8
VP5	1182	4.49	0.843	1338.9	1406.0	0.996	0.44	148.0
CA1	1083	5.23	0.902	1267.3	1326.4	0.995	0.519	135.6
CA2	997	3.47	0.692	1166.6	1227.1	0.997	0.348	117.0
CA3	1338	5.56	0.878	1509.3	1598.6	0.995	0.535	154.2
CA4	1303	6.18	0.944	1429.3	1508.5	0.995	0.597	156.6
CA5	1237	5.14	0.874	1375.0	1451.2	0.996	0.501	152.8
CE1	1235	4.87	0.848	1377.2	1455.6	0.996	0.474	143.2
CE2	1494	7.74	0.988	1608.3	1676.5	0.996	0.734	163.7
CE3	1397	6.25	0.924	1539.6	1602.1	0.995	0.598	159.6
CE4	1287	4.9	0.783	1456.0	1550.6	0.995	0.474	156.9
CE5	1230	5.49	0.914	1388.6	1459.7	0.996	0.535	142.5
CN1	1305	5.64	0.905	1450.7	1521.2	0.995	0.545	154.5
CN2	1379	6.01	0.934	1544.4	1605.1	0.995	0.576	159.8
CN3	1815	8.05	0.986	1872.6	1958.2	0.995	0.744	226.9
CN4	1869	7.82	0.977	1937.4	2038.2	0.994	0.719	213.6
DD1	1081	4.6	0.855	1299.4	1344.8	0.996	0.457	125.1
DD2	1432	7.33	0.979	1549.0	1592.1	0.997	0.699	150.4
DD3	1120	4.06	0.791	1293.3	1346.9	0.996	0.401	139.1
DD4	1460	6.88	0.967	1607.0	1675.7	0.995	0.655	160.8
ED1	1200	4.75	0.86	1397.6	1440.0	0.996	0.464	131.1

ED2	1115	3.98	0.793	1305.2	1372.5	0.996	0.393	139.3
ED3	1126	4.48	0.841	1309.1	1376.9	0.996	0.442	145.4
ED4	1135	4.69	0.891	1383.3	1446.2	0.995	0.462	135.1
ED5	1066	4.53	0.891	1285.9	1357.2	0.996	0.45	128.3

Notes: Sobs = observed OTUs; Chao/ACE = richness estimators; Pielou = evenness; Goods_cov (OTU) = Good's coverage at the OTU level; PD = Faith's phylogenetic diversity.

Table S3. PERMANOVA and ANOSIM results based on Bray–Curtis dissimilarity.

Distance	Df	SumsOfSqs	MeanSqs	F	R^2	p
Bray–Curtis	2	1.3314	0.6657	3.131	0.1075	0.001

Distance	R	p	Permutations
Bray–Curtis	0.1571	0.001	—

Notes: Distances computed on relative abundances. PERMANOVA: adonis2 (permutation test); ANOSIM permutations not reported in the source sheet (—).

Table S4. LEfSe-derived microbial biomarkers (LDA ≥ 3.5, log10) across host families.

Rank	Taxon	Lineage	Enriched_in	LDA (log10)	p	FDR
Class	Alphaproteobacteria	Bacteria.Proteobacteria.Alphaproteobacteria	BOV	3.87	0.0154	0.0204
Family	Moraxellaceae	Bacteria.Proteobacteria.Gammaproteobacteria.Pseudomonadales.Moraxellaceae	BOV	5.00	0.0122	0.0171
Family	Rhodobacteraceae	Bacteria.Proteobacteria.Alphaproteobacteria.Rhodobacterales.Rhodobacteraceae	BOV	3.60	0.00174	0.00458
Genus	Acinetobacter	Bacteria.Proteobacteria.Gammaproteobacteria.Pseudomonadales.Moraxellaceae.Acinetobacter	BOV	5.01	0.01	0.0151
Genus	Sphingobacterium	Bacteria.Bacteroidetes.Bacteroidia.Sphingobacteriales.Sphingobacteriaceae.Sphingobacterium	BOV	4.28	0.00084	0.0027
Order	Rhodobacterales	Bacteria.Proteobacteria.Alphaproteobacteria.Rhodobacterales	BOV	3.60	0.00174	0.0046
Species	Acinetobacter_lwoffii	Bacteria.Proteobacteria.Gammaproteobacteria.Pseudomonadales.Moraxellaceae.Acinetobacter.Acinetobacter_lwoffii	BOV	5.01	0.00668	0.0115
Class	Verrucomicrobiae	Bacteria.Verrucomicrobia.Verrucomicrobiae	CAM	3.67	0.00725	0.0116
Family	Christensenellaceae	Bacteria.Firmicutes.Clostridia.Clostridiales.Christensenellaceae	CAM	3.87	0.0123	0.0172
Family	Akkermansiaceae	Bacteria.Verrucomicrobia.Verrucomicrobiae.Verrucomicrobiales.Akkermansiaceae	CAM	3.67	0.00684	0.0116

Genus	Rummeliibacillus	Bacteria.Firmicutes.Bacilli.Bacillales.Planococcaceae.Rummeliibacillus	CAM	3.88	0.00251	0.0058
Genus	Christensenellaceae_R_7_group	Bacteria.Firmicutes.Clostridia.Clostridiales.Christensenellaceae.Christensenellaceae_R_7_group	CAM	3.86	0.0108	0.0158
Genus	Jeotgalicoccus	Bacteria.Firmicutes.Bacilli.Bacillales.Staphylococcaceae.Jeotgalicoccus	CAM	3.79	0.00696	0.0116
Genus	Atopostipes	Bacteria.Firmicutes.Bacilli.Lactobacillales.Carnobacteriaceae.Atopostipes	CAM	3.70	0.00189	0.0048
Genus	Proteus	Bacteria.Proteobacteria.Gammaproteobacteria.Enterobacteriales.Enterobacteriaceae.Proteus	CAM	3.67	0.00029	0.0012
Genus	Akkermansia	Bacteria.Verrucomicrobia.Verrucomicrobiae.Verrucomicrobiales.Akkermansiaceae.Akkermansia	CAM	3.67	0.00684	0.0116
Order	Verrucomicrobiales	Bacteria.Verrucomicrobia.Verrucomicrobiae.Verrucomicrobiales	CAM	3.67	0.00654	0.0114
Phylum	Verrucomicrobia	Bacteria.Verrucomicrobia	CAM	3.67	0.00725	0.0116
Species	Rummeliibacillus_pycnus	Bacteria.Firmicutes.Bacilli.Bacillales.Planococcaceae.Rummeliibacillus.Rummeliibacillus_pycnus	CAM	3.88	0.00251	0.0058
Species	Proteus_mirabilis	Bacteria.Proteobacteria.Gammaproteobacteria.Enterobacteriales.Enterobacteriaceae.Proteus.Proteus_mirabilis	CAM	3.67	0.00029	0.0012
Class	Spirochaetia	Bacteria.Spirochaetes.Spirochaetia	CER	3.93	1.85e-06	3.01e-05
Family	Rikenellaceae	Bacteria.Bacteroidetes.Bacteroidia.Bacteroidales.	CER	4.16	0.00074	0.0025

		Rikenellaceae				
Family	Peptostreptococcaceae	Bacteria.Firmicutes.Clostridia.Clostridiales.Peptostreptococcaceae	CER	3.95	9.22e-06	8.36e-05
Family	Spirochaetaceae	Bacteria.Spirochaetes.Spirochaetia.Spirochaetales.Spirochaetaceae	CER	3.93	1.85e-06	3.01e-05
Family	Clostridiaceae_1	Bacteria.Firmicutes.Clostridia.Clostridiales.Clostridiaceae_1	CER	3.81	0.00246	0.0058
Family	Bifidobacteriaceae	Bacteria.Actinobacteria.Actinobacteria.Bifidobacteriales.Bifidobacteriaceae	CER	3.80	0.0127	0.0175
Family	Streptococcaceae	Bacteria.Firmicutes.Bacilli.Lactobacillales.Streptococcaceae	CER	3.64	0.00216	0.0053
Family	Bacteroidaceae	Bacteria.Bacteroidetes.Bacteroidia.Bacteroidales.Bacteroidaceae	CER	3.62	0.0344	0.0381
Genus	Rikenellaceae_RC9_gut_group	Bacteria.Bacteroidetes.Bacteroidia.Bacteroidales.Rikenellaceae.Rikenellaceae_RC9_gut_group	CER	4.03	0.00034	0.0013
Genus	Ruminococcaceae_UCG_005	Bacteria.Firmicutes.Clostridia.Clostridiales.Ruminococcaceae.Ruminococcaceae_UCG_005	CER	3.96	0.0224	0.0276
Genus	Treponema_2	Bacteria.Spirochaetes.Spirochaetia.Spirochaetales.Spirochaetaceae.Treponema_2	CER	3.92	2.41e-06	3.18e-05
Genus	Clostridium_sensu_stricto_1	Bacteria.Firmicutes.Clostridia.Clostridiales.Clostridiaceae_1.Clostridium_sensu_stricto_1	CER	3.74	0.00069	0.0024
Genus	Paeniclostridium	Bacteria.Firmicutes.Clostridia.Clostridiales.Peptostreptococcaceae.Paeniclo	CER	3.68	2.24e-06	3.18e-05

		stridium				
Genus	Bacteroides	Bacteria.Bacteroidetes.Bacteroidia.Bacteroidales.Bacteroidaceae.Bacteroides	CER	3.62	0.0344	0.0381
Genus	Bacillus	Bacteria.Firmicutes.Bacilli.Bacillales.Bacillaceae.Bacillus	CER	3.61	0.00441	0.0084
Genus	Streptococcus	Bacteria.Firmicutes.Bacilli.Lactobacillales.Streptococcaceae.Streptococcus	CER	3.59	0.00126	0.0037
Genus	Romboutsia	Bacteria.Firmicutes.Clostridia.Clostridiales.Peptostreptococcaceae.Romboutsia	CER	3.53	0.00053	0.0019
Order	Bacteroidales	Bacteria.Bacteroidetes.Bacteroidia.Bacteroidales	CER	4.48	0.00295	0.0065
Order	Spirochaetales	Bacteria.Spirochaetes.Spirochaetia.Spirochaetales	CER	3.93	1.85e-06	3.01e-05
Order	Bifidobacteriales	Bacteria.Actinobacteria.Actinobacteria.Bifidobacteriales	CER	3.80	0.0127	0.0175
Phylum	Spirochaetes	Bacteria.Spirochaetes	CER	3.93	1.85e-06	3.01e-05

Notes: LDA scores shown to two decimals; p and FDR adjusted using Benjamini–Hochberg.

Table S5. KEGG Level 2 functional profiles (PICRUSt2) summarized by host family.

Level_1	Level_2	BOV (%)	CER (%)	CAM (%)	Highest_in	Δ(max-min) (pp)
Cellular Processes	Cell motility	1.65	2.17	2.16	CER	0.52
Cellular Processes	Transport and catabolism	0.44	0.41	0.42	BOV	0.03
Cellular Processes	Cellular community - prokaryotes	0.16	0.17	0.18	CAM	0.02
Cellular Processes	Cell growth and death	1.20	1.21	1.21	CER	0.01
Environmental Information Processing	Membrane transport	1.79	1.83	1.85	CAM	0.06
Environmental Information Processing	Signal transduction	0.46	0.49	0.51	CAM	0.05
Environmental Information Processing	Signaling molecules and interaction	0.00	0.00	0.00	CAM	0.00
Genetic Information Processing	Replication and repair	5.00	5.27	5.17	CER	0.28
Genetic Information Processing	Translation	2.75	2.91	2.81	CER	0.16
Genetic Information Processing	Transcription	0.78	0.92	0.87	CER	0.14
Genetic Information Processing	Folding, sorting and degradation	3.21	3.18	3.16	BOV	0.04
Human Diseases	Infectious diseases	0.19	0.27	0.25	CER	0.08
Human Diseases	Cancers	0.01	0.00	0.01	BOV	0.01
Human Diseases	Neurodegenerative diseases	0.01	0.02	0.01	CER	0.00
Human Diseases	Cardiovascular diseases	0.00	0.00	0.00	CAM	0.00
Human Diseases	Immune diseases	0.00	0.00	0.00	BOV	0.00
Metabolism	Xenobiotics biodegradation and	8.46	7.44	7.62	BOV	1.02

	metabolism					
Metabolism	Carbohydrate metabolism	12.23	12.72	12.29	CER	0.48
Metabolism	Metabolism of terpenoids and polyketides	9.08	8.78	8.62	BOV	0.46
Metabolism	Lipid metabolism	8.09	7.72	7.90	BOV	0.37
Metabolism	Metabolism of cofactors and vitamins	12.38	12.27	12.61	CAM	0.33
Metabolism	Metabolism of other amino acids	7.17	7.14	7.40	CAM	0.26
Metabolism	Glycan biosynthesis and metabolism	2.89	2.86	2.71	BOV	0.18
Metabolism	Amino acid metabolism	13.18	13.20	13.36	CAM	0.17
Metabolism	Nucleotide metabolism	1.66	1.78	1.73	CER	0.12
Metabolism	Biosynthesis of other secondary metabolites	1.60	1.64	1.58	CER	0.06
Metabolism	Energy metabolism	5.26	5.26	5.24	BOV	0.02
Organismal Systems	Endocrine system	0.08	0.09	0.07	CER	0.01
Organismal Systems	Environmental adaptation	0.18	0.18	0.18	CAM	0.01
Organismal Systems	Digestive system	0.02	0.02	0.02	CAM	0.00
Organismal Systems	Immune system	0.05	0.05	0.05	CAM	0.00
Organismal Systems	Development	0.00	0.00	0.00	BOV	0.00
Organismal Systems	Excretory system	0.00	0.00	0.00	CAM	0.00

Notes: Values are percentages within each family (sum of KEGG L2 pathways = 100% per family).
