# Supplementary figures and images for: Host Phylogeny Shapes Gut Microbiota and Predicted Functions in Captive Artiodactyls
Source: Microorganisms. 2025 Sep 25;13(10):2250. doi: 10.3390/microorganisms13102250 (PMC12566460; doi:10.3390/microorganisms13102250)

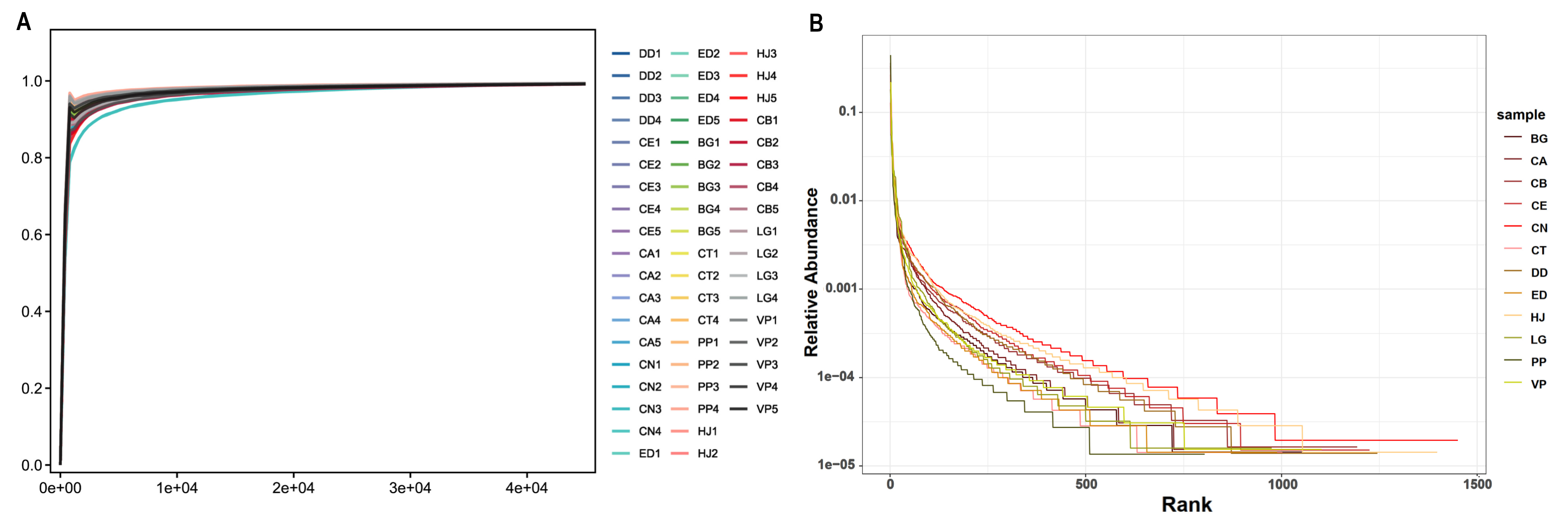

Supplement: Supplementary file 1 [file microorganisms-13-02250-s001.zip › Figure S1.png]

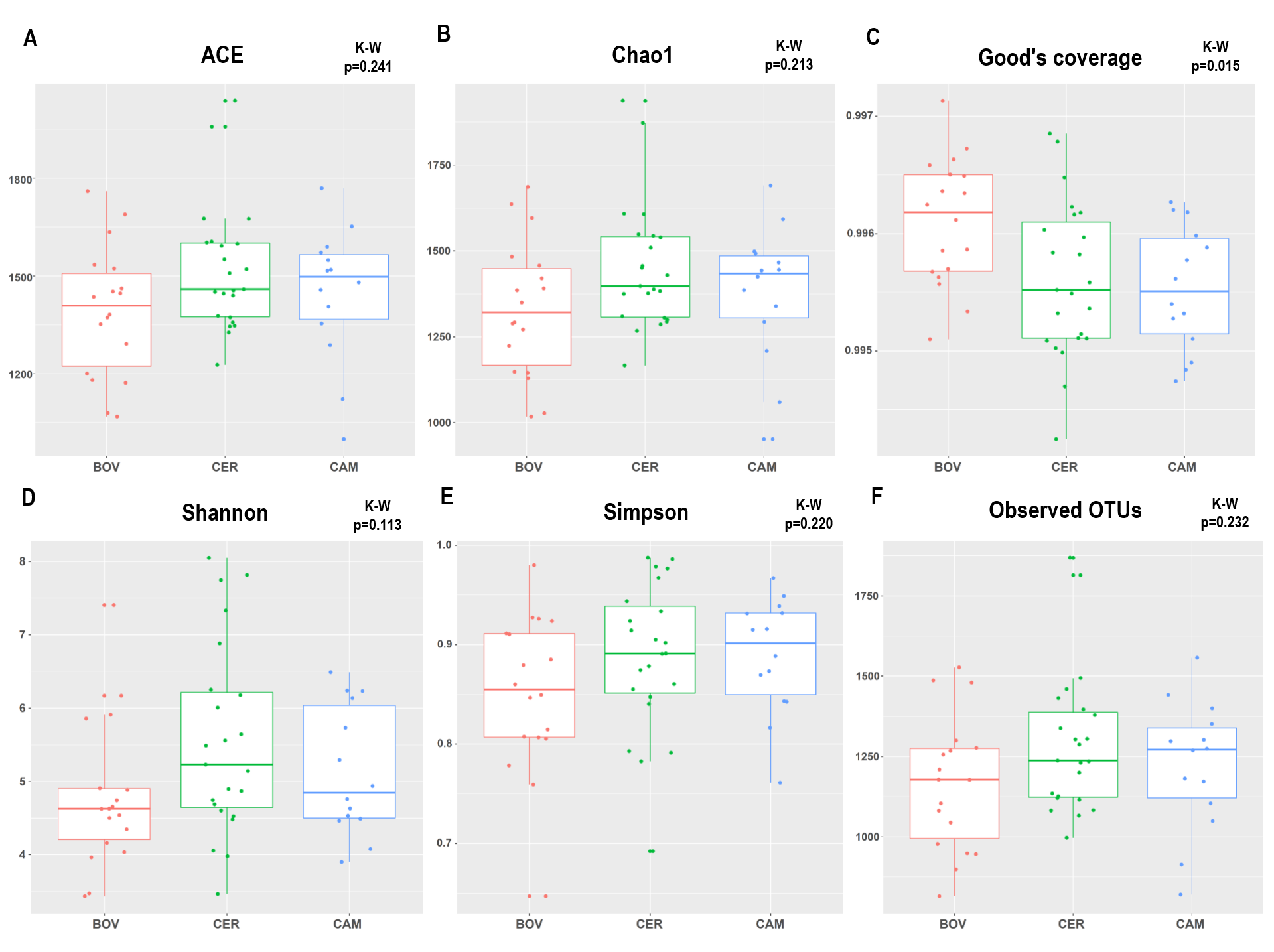

Supplement: Supplementary file 1 [file microorganisms-13-02250-s001.zip › Figure S2.png]

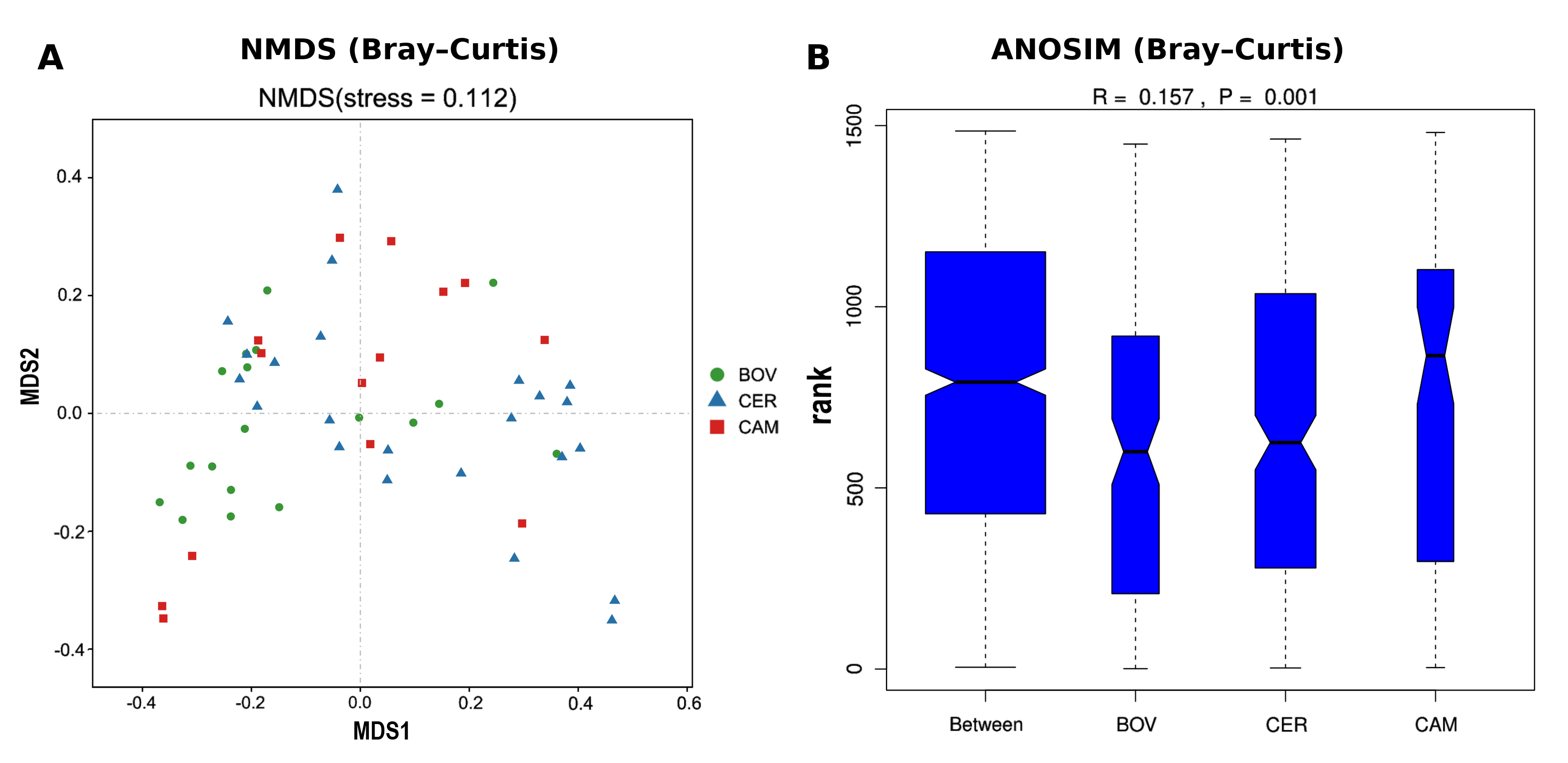

Supplement: Supplementary file 1 [file microorganisms-13-02250-s001.zip › Figure S3.png]

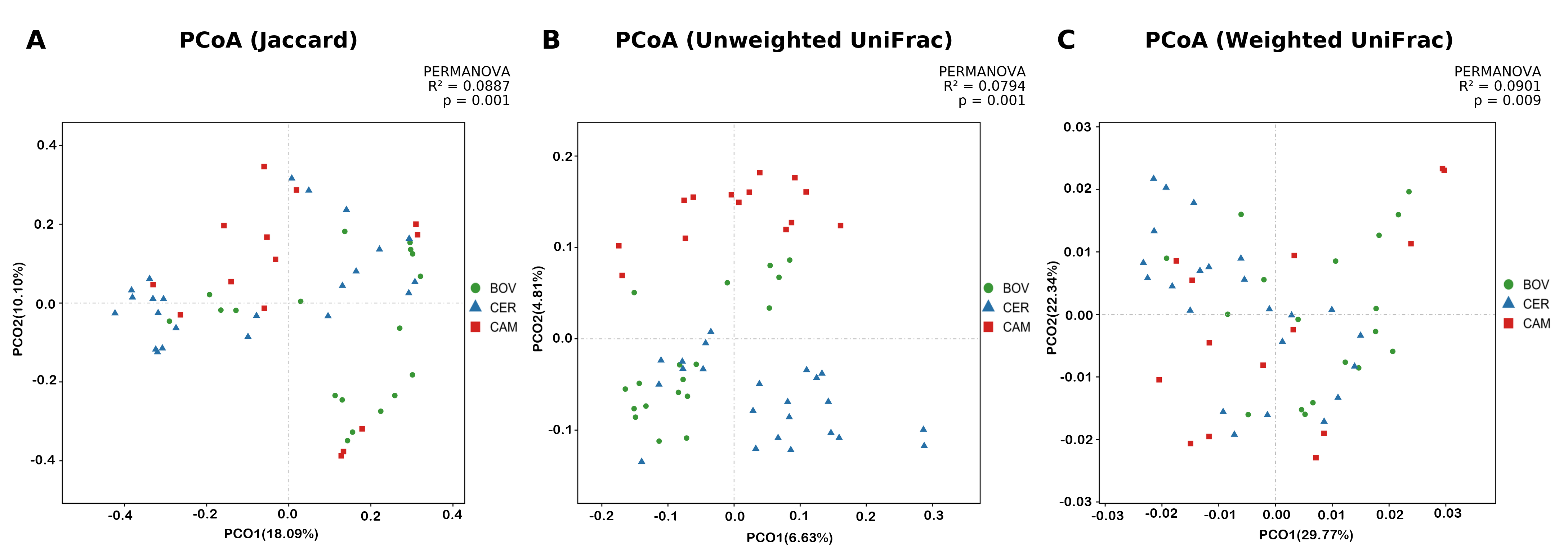

Supplement: Supplementary file 1 [file microorganisms-13-02250-s001.zip › Figure S4.png]
